# Supplementary material for: Development and validation of a predictive model for critical illness in adult patients requiring hospitalization for COVID-19
Source: PLoS One. 2021 Mar 19;16(3):e0248891. doi: 10.1371/journal.pone.0248891 (PMC7978341; doi:10.1371/journal.pone.0248891)
Supplement: S1 Appendix — (DOCX) [file pone.0248891.s001.docx]

**S1 Appendix.**

**Appendix A: Variables Considered for Backwards Stepwise Selection for Models Predicting Intensive Care Unit Status**

- Age
- Female
- Black race
- White race
- Temperature
- Oxygen saturation
- Body mass index
- Chronic obstructive pulmonary disease (COPD) or asthma
- Coronary artery disease
- Chronic kidney disease
- Diabetes mellitus
- Hypertension
- Serum lactate dehydrogenase
- Serum ferritin
- Serum D-dimer
- Serum C-reactive protein
- Absolute lymphocyte count
- Age X Race
- Age X Diabetes mellitus
- Age X Coronary artery disease
- Age X Chronic kidney disease
- Age X Hypertension
- Hypertension X Diabetes mellitus
- Serum ferritin X Serum Lactate dehydrogenase
- Serum lactate dehydrogenase x Serum D-dimer
- Serum lactate dehydrogenase x Serum C-reactive protein
- Serum ferritin x Serum D-dimer

*All variables with correlations exceeding 0.20 (absolute value) were included as potential interactions. The reference group for race is Other Race. The reference group for sex is Male.*

**Appendix B: Correlations Between Demographic Predictor Variable Candidates.**

**
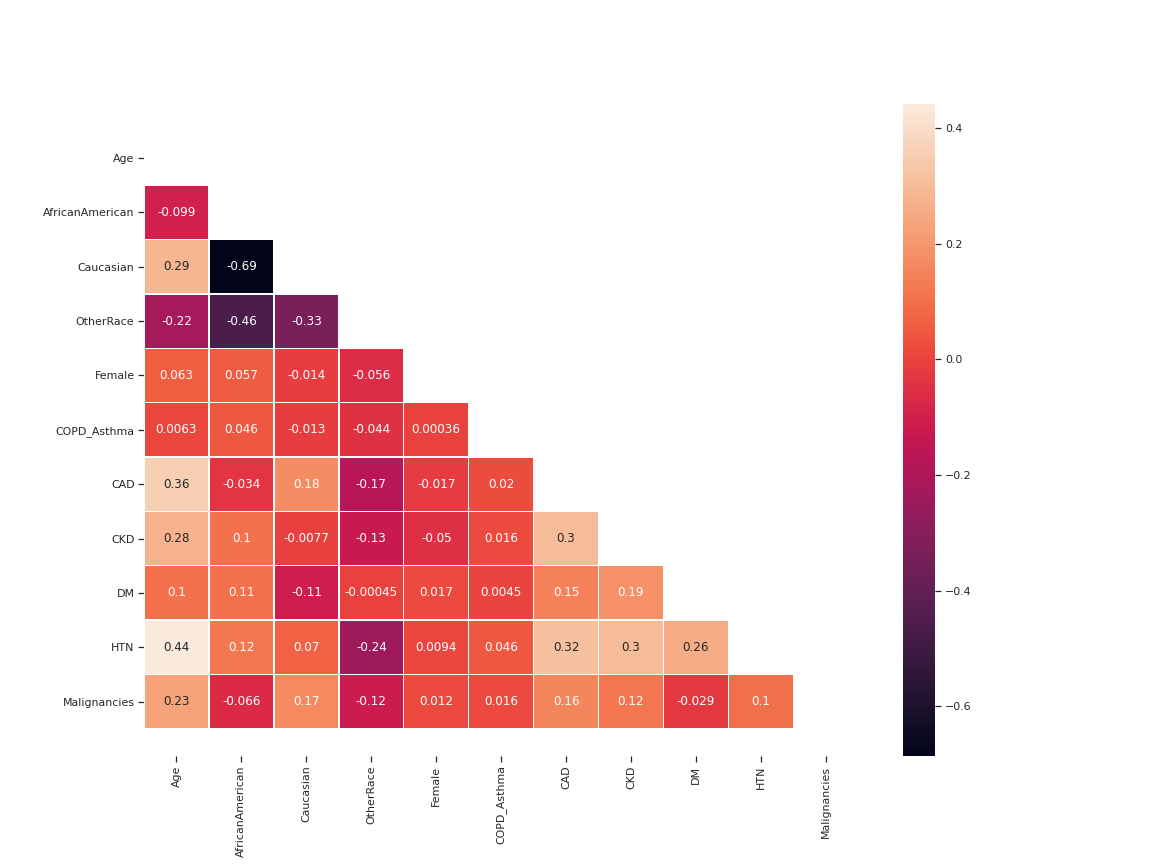
**

**Appendix C: Correlations Between Laboratory Predictor Variable Candidates.**

**
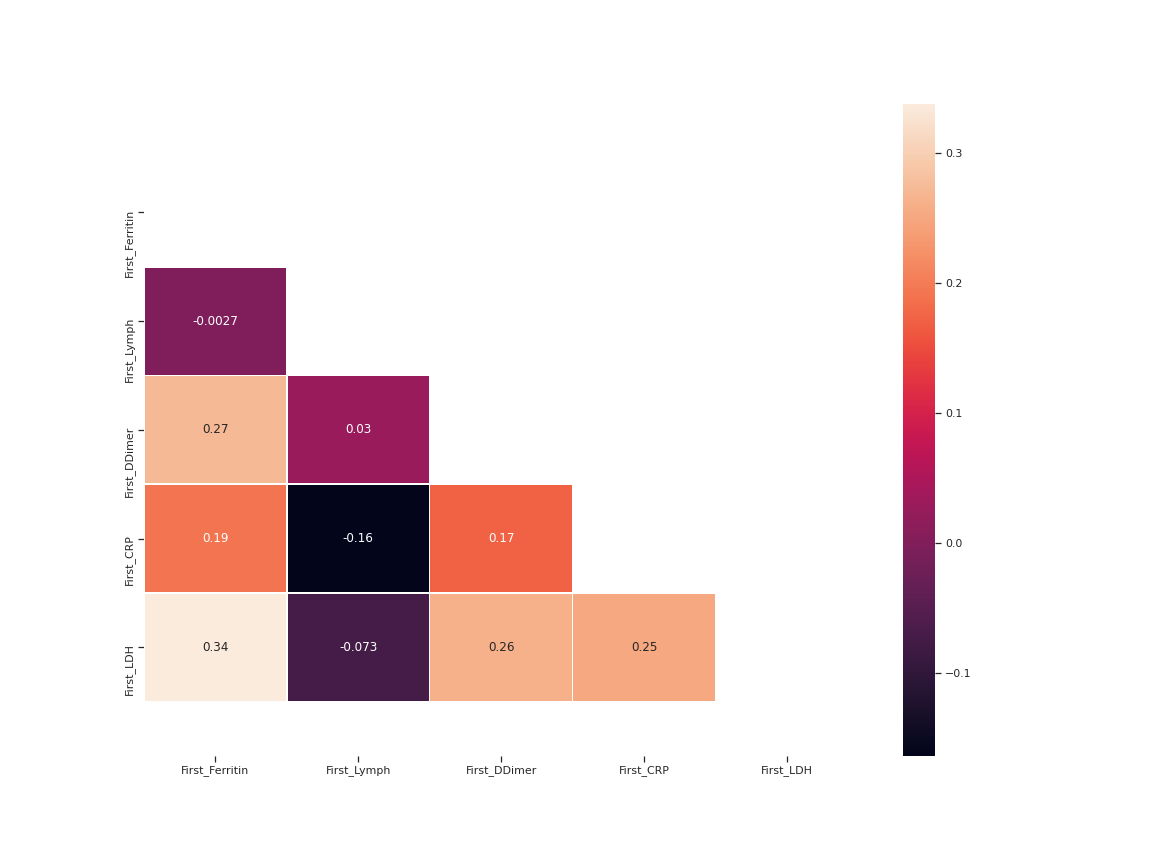
**

**Appendix D: Missing Data**

| **Variable** | **%Missing** |
| --- | --- |
| Age | **0%** |
| Sex | **0%** |
| BMI | **0%** |
| Race | **0.5%** |
| Temperature | **0.7%** |
| Oxygen Saturation | **0.7%** |
| CRP | **9.8%** |
| LDH | **9.7%** |
| Ferritin | **4.2%** |
| D-dimer | **9.3%** |
| Absolute lymphocyte count | **0.2%** |

**Appendix E: Probability score with corresponding Sensitivity, Specificity, Positive Predictive Value, Negative Predictive Value, Positive Likelihood Ratio, Negative Likelihood Ratio and Youden Index**

| **Probability** | **Sensitivity** | **Specificity** | **PPV** | **NPV** | **LR (Pos)** | **LP (Neg)** | **Youden** |
| --- | --- | --- | --- | --- | --- | --- | --- |
| **10%** | **100%** | **1%** | **29%** | **100%** | **1.01** | **0.00** | **0.01** |
| **20%** | **95%** | **27%** | **34%** | **94%** | **1.31** | **0.17** | **0.23** |
| **30%** | **82%** | **53%** | **41%** | **88%** | **1.77** | **0.33** | **0.36** |
| **40%** | **67%** | **70%** | **47%** | **84%** | **2.22** | **0.48** | **0.37** |
| **50%** | **55%** | **84%** | **58%** | **82%** | **3.39** | **0.54** | **0.39** |
| **60%** | **40%** | **91%** | **64%** | **79%** | **4.51** | **0.66** | **0.31** |
| **70%** | **31%** | **95%** | **71%** | **77%** | **6.31** | **0.73** | **0.26** |
| **80%** | **18%** | **98%** | **81%** | **75%** | **10.91** | **0.83** | **0.17** |
| **90%** | **8%** | **99%** | **80%** | **73%** | **9.98** | **0.92** | **0.07** |

**Appendix F: Comparison of variables by patients ICU transfer status**

| **Variable** | **Not transferred to ICU**  **(Mean / Percent)** | **Transferred to ICU**  **(Mean / Percent)** |
| --- | --- | --- |
| **Temperature F** | **97.7** | **98.8** |
| **Oxygen saturation (%)** | **95.1** | **94.5** |
| **C-reactive protein (mg/dl)** | **7.8** | **13.1** |
| **Lactate dehydrogenase (mg/ml)** | **323.1** | **455** |
| **Ferritin (ng/dl)** | **788** | **1378** |
| **D-dimer (ng/dl)** | **1029** | **2127** |
| **Absolute lymphocyte count** | **1.2** | **1.07** |
| **Age** | **58.7** | **61.3** |
| **BMI** | **31.4** | **32.8** |
| **Black/African-American** | **45.6%** | **49.3%** |
| **White/Caucasian** | **35%** | **31.2%** |
| **Other race** | **19.3%** | **19.4%** |
| **Hypertension** | **42.9%** | **29%** |
| **Diabetes Mellitus** | **29.5%** | **25.2%** |
| **Coronary Artery Disease** | **16.2%** | **12.5%** |
| **Malignancies** | **7.8%** | **4.5%** |
| **COPD/Asthma** | **1.1%** | **1.3%** |

**Appendix G: Completed Checklist to Indicate Adherence to the TRIPOD Reporting Guideline for Studies Developing and Validating Multivariable Predictive Models**

| **Section/Topic** | **Item** |  | **Checklist Item** | **Page** |
| --- | --- | --- | --- | --- |
| **Title and abstract** | | | | |
| Title | 1 | D;V | Identify the study as developing and/or validating a multivariable prediction model, the target population, and the outcome to be predicted. | 1 |
| Abstract | 2 | D;V | Provide a summary of objectives, study design, setting, participants, sample size, predictors, outcome, statistical analysis, results, and conclusions. | 2 |
| **Introduction** | | | | |
| Background and objectives | 3a | D;V | Explain the medical context (including whether diagnostic or prognostic) and rationale for developing or validating the multivariable prediction model, including references to existing models. | 3-4 |
|  | 3b | D;V | Specify the objectives, including whether the study describes the development or validation of the model or both. | 4 |
| **Methods** | | | | |
| Source of data | 4a | D;V | Describe the study design or source of data (e.g., randomized trial, cohort, or registry data), separately for the development and validation data sets, if applicable. | 4 |
|  | 4b | D;V | Specify the key study dates, including start of accrual; end of accrual; and, if applicable, end of follow-up. | 4 |
| Participants | 5a | D;V | Specify key elements of the study setting (e.g., primary care, secondary care, general population) including number and location of centres. | 4 |
|  | 5b | D;V | Describe eligibility criteria for participants. | 4 |
|  | 5c | D;V | Give details of treatments received, if relevant. | NA |
| Outcome | 6a | D;V | Clearly define the outcome that is predicted by the prediction model, including how and when assessed. | 5 |
|  | 6b | D;V | Report any actions to blind assessment of the outcome to be predicted. | NA |
| Predictors | 7a | D;V | Clearly define all predictors used in developing or validating the multivariable prediction model, including how and when they were measured. | 5 |
|  | 7b | D;V | Report any actions to blind assessment of predictors for the outcome and other predictors. | NA |
| Sample size | 8 | D;V | Explain how the study size was arrived at. | 4-5 |
| Missing data | 9 | D;V | Describe how missing data were handled (e.g., complete-case analysis, single imputation, multiple imputation) with details of any imputation method. | 5 |
| Statistical analysis methods | 10a | D | Describe how predictors were handled in the analyses. | 5-6 |
|  | 10b | D | Specify type of model, all model-building procedures (including any predictor selection), and method for internal validation. | 5-6 |
|  | 10c | V | For validation, describe how the predictions were calculated. | 5-6 |
|  | 10d | D;V | Specify all measures used to assess model performance and, if relevant, to compare multiple models. | 5-6 |
|  | 10e | V | Describe any model updating (e.g., recalibration) arising from the validation, if done. | NA |
| Risk groups | 11 | D;V | Provide details on how risk groups were created, if done. | NA |
| Development vs. validation | 12 | V | For validation, identify any differences from the development data in setting, eligibility criteria, outcome, and predictors. | 4 |
| **Results** | | | | |
| Participants | 13a | D;V | Describe the flow of participants through the study, including the number of participants with and without the outcome and, if applicable, a summary of the follow-up time. A diagram may be helpful. | 6-7 |
|  | 13b | D;V | Describe the characteristics of the participants (basic demographics, clinical features, available predictors), including the number of participants with missing data for predictors and outcome. | 6-7  Appendix |
|  | 13c | V | For validation, show a comparison with the development data of the distribution of important variables (demographics, predictors and outcome). | 6 |
| Model development | 14a | D | Specify the number of participants and outcome events in each analysis. | 7-8 |
|  | 14b | D | If done, report the unadjusted association between each candidate predictor and outcome. | Appendix |
| Model specification | 15a | D | Present the full prediction model to allow predictions for individuals (i.e., all regression coefficients, and model intercept or baseline survival at a given time point). | 8-9 |
|  | 15b | D | Explain how to the use the prediction model. | 10-12 |
| Model performance | 16 | D;V | Report performance measures (with CIs) for the prediction model. | 9 |
| Model-updating | 17 | V | If done, report the results from any model updating (i.e., model specification, model performance). | NA |
| **Discussion** | | | | |
| Limitations | 18 | D;V | Discuss any limitations of the study (such as nonrepresentative sample, few events per predictor, missing data). | 11-12 |
| Interpretation | 19a | V | For validation, discuss the results with reference to performance in the development data, and any other validation data. | 11 |
|  | 19b | D;V | Give an overall interpretation of the results, considering objectives, limitations, results from similar studies, and other relevant evidence. | 12 |
| Implications | 20 | D;V | Discuss the potential clinical use of the model and implications for future research. | 12-13 |
| **Other information** | | | | |
| Supplementary information | 21 | D;V | Provide information about the availability of supplementary resources, such as study protocol, Web calculator, and data sets. | 10 |
| Funding | 22 | D;V | Give the source of funding and the role of the funders for the present study. | 1 |

*Items relevant only to the development of a prediction model are denoted by D, items relating solely to a validation of a prediction model are denoted by V, and items relating to both are denoted D;V.

Abbreviations: NA = not applicable
