# Supplementary material for: Development and validation of a predictive model for critical illness in adult patients requiring hospitalization for COVID-19
Source: PLoS One. 2021 Mar 19;16(3):e0248891. doi: 10.1371/journal.pone.0248891 (PMC7978341; doi:10.1371/journal.pone.0248891)
Supplement: Attachment — Submitted filename: PlosResponseReviewers.docx [file pone.0248891.s004.docx]

Response to Reviewers

Reviewer #1: This manuscript is very clearly written and the objective is very well defined: to set a predicted risk score for ICU admission. I would say that external validity for this score is questionable, but authors have defined that the goal is to build a predictive score for a defined population. In this sense, I think statistical analysis was appropriate. I appreciate the low amount of missing data in such a large population.

Response: We thank the reviewer for their comments.

My concerns are quoted by authors in the discussion: the comorbidities the authors found to be related with ICU admission in this population are not the ones we have seen to be ICU admission predictors in literature so far. I don't understand why authors have chosen only oxygen saturation to account for respiratory pattern; I would also include respiratory frequency and need for any ventilatory support out of the ICU - these are main predictors of need for intensive care and mechanical ventilation. It's not clear whether patients were in room air or if they are already using some kind of oxygen support.

Response: We appreciate the comment. As mentioned in our discussion, the model was developed quickly in response to local need during the COVID-19 surge, when hospitals in our health system were at crisis capacity. The calculator was intended to be used to triage hospital admissions during the surge. As outlined in the Methods section, we chose to use oxygen saturation at **room air** measured at admission because it would give us the most accurate estimation of need for respiratory support.

In addition, the authors present median BMI, but don't account for BMI in their score - this is strange, since high BMI is a predictor of ICU admission for COVID-19 in many populations across the world.

Response: We included BMI among the variables collected for the model. We have added to the Discussion that, somewhat surprisingly, BMI did not emerge as an independent risk factor. We discuss that this was likely because our study included only hospitalized patients and a majority of patients needing hospitalization in our cohort had a high BMI.

I would also ask for data regarding patients who were and were not admitted to the ICU. A table comparing data (such as presented in tables 1 and 2) between those admitted vs. not admitted to the ICU is key. I understand that CKD, CAD, CRP and LDH are predictors of ICU admission in this sample, but I would like to know median values for each one in those admitted vs. not admitted to ICU.

Response: We have included a table comparing data between those admitted to the ICU vs those not admitted to the ICU (Appendix F).

Reviewer #2: Thanks for your invitation. Prediction for severe or critical illness of COVID-19 is an important issue which are attracting increasing attention for clinicians and researchers. The authors developed and validated a simple and easily available tool to predict ICU service for in-hospital patients with COVID-19. Followings are the comments to the authors that should be considered.

Response: We thank the reviewer for their comments.

1. There are emerging articles reported the predictors or prognostic models for severe or critical illness in patients with COVID-19. Did you systematically summarize the reported predictors or variables that should be included in your prediction model? I am afraid a wide array of predictors, such as neutrophil to lymphocyte ratio, blood glucose level, serum creatinine, procalcitonin, troponin and so on, were missed in your model.1-3 In addition, many of them are commonly available at most acute care facilities. So why those potential predictors were not considered in your model?

Response: Thanks for this comment. We agree that the model does not include or consider all possible variables. As explained in our response to Reviewer 1 (above), the model was created quickly in response to a local need. We have acknowledged in our Limitations that due to the constant evolving nature of COVID-19 research, we included only a finite number of variables and characteristics that were implicated in prediction of severe disease during the initial months of the pandemic. A reassuring and perhaps attractive aspect of our model is that despite its parsimony, its performance (measured using a standard approach such as the area under the curve (AUC) is comparable to other available and more expansive models.

2. What is the definition and method for measurement of outcome to be predicted? What is the criteria of patients transfer to ICU service?

Response: The Methods section defines the outcome of interest as transfer to the ICU service (irrespective of the patient’s physical location). We have added clarification that the criteria for transfer to ICU service remained the same as it is for non-COVID patients, as determined by the on-call Critical Care provider.

3. This is a retrospective study based on data from health care system. So there is more potential risk of bias compared with perspective study. What methods have you used to minimize the risk of bias in the present study?

Response: We appreciate the comment about potential bias. As described in the Methods section, we paid careful attention to applying our eligibility criteria and assessing the outcome consistently. Our multivariable model adequately accounts for expected confounders of interest.

4. I noted that you had compared your work with similar studies in discussion section. Many large scale, high-quality articles had published in top journals, and more and more similar articles are emerging. So, except for easy availability and feasibility, what are the main advantages of your model compared with other prediction model? What’s new in your work?

Response: We agree that there are a number of available predictive models. We do not claim that our model is the best or that the study is entirely novel. We have bolstered the Discussion to point out our model’s strengths acknowledge its limitations in the context of other models. The main advantages are our study’s large sample size, representation of diverse populations, and the model’s performance, applicability, easy availability, and use of easily available variables.

5. This is a single center study. There was no external validation. The prediction model is feasible and useful for local need. However, the generalizability of this model to external population might be unclear. Did you have any plan to validate your model in external data?

Response: We have acknowledged in the Discussion that the model’s applicability across settings is a limitation. We are careful in the language of the Discussion to not suggest that it is generalizable. External validation was beyond the scope of the current study.

6. The present study did not mention treatments patients received. Some of them may be protective factor in your model.

Response: We agree that treatments after admission may be protective. However, the current study considered patients admitted between March 1, 2020 and July 10, 2020. While remdesivir and dexamethasone are now considered standard of care, these agents were not freely available to our patients during our study period. Early in the pandemic, use of corticosteroids was discouraged. It was only in mid-July that data from the RECOVERY trial showed a mortality benefit in patients with hypoxia due to COVID-19. Remdesivir was FDA approved for the treatment of COVID-19 only in October 2020. Further our risk calculator was developed using variables on admission and therefore predicts before the initiation of in-patient treatments, such as remdesivir and dexamethasone that are now considered standard of care for hospitalized patients.

7. Except for logistic regression method, it will be better to compare the accuracy with other multivariable models.

Response: The logistic regression model was developed in accordance with an *a priori* analysis plan that employed backwards stepwise variable selection. Comparing the model development approach with other statistical models is an interesting statistical methods project but is beyond the scope of the current work.

8. Some references are incomplete. (line 344-346, 358-370)
REFERENCES
[1] Petrilli C M, Jones S A, Yang J, et al. Factors associated with hospital admission and critical illness among 5279 people with coronavirus disease 2019 in New York City: prospective cohort study[J]. Bmj, 2020, 369.

Response: We have incorporated this reference per the reviewer’s suggestion.

[2] Zhu L, She Z G, Cheng X, et al. Association of blood glucose control and outcomes in patients with COVID-19 and pre-existing type 2 diabetes[J]. Cell metabolism, 2020, 31(6): 1068-1077. e3.

Response: We have incorporated this reference per the reviewer’s suggestion.

[3] Liang W, Liang H, Ou L, et al. Development and validation of a clinical risk score to predict the occurrence of critical illness in hospitalized patients with COVID-19[J]. JAMA internal medicine, 2020, 180(8): 1081-1089.

Response: We have incorporated this reference per the reviewer’s suggestion.
